# Nakajo-Nishimura Syndrome: The First African Case

**DOI:** 10.31138/mjr.34.2.262

**Published:** 2023-06-30

**Authors:** Nacif Eddine Ghodbane, Ali Mecibah, Zohra Merzougui, Halima Zerguine, Zineb Akakba, Samy Slimani

**Affiliations:** 1 Department of Otorhinolaryngology Head and Neck Surgery EPH Houas Salah ORL Clinic, Benboulaid Batna, Algeria,; 2 Department of Pediatrics CHU Benflis Touhami, allées Mohamed boudiaf, Batna, Algeria,; 3 Department of Cardiology CHU Mustapha Pacha, Algiers, Algeria,; 4 Rheumatology Clinic, Batna, Algeria

**Keywords:** Nakajo-Nishimura syndrome, hereditary, Africa, Algeria, chronic atypical neutrophilic dermatosis with lipodystrophy and elevated temperature

## Abstract

Nakajo-Nishimura syndrome is a hereditary autoinflammatory disorder caused by an autosomal recessive homozygous mutation of the PSMB8 gene, which encodes the immunoproteasome subunit beta 5i. The clinical manifestations of NNS are mainly pernio-like skin rashes, nodular erythema, lipodystrophy, clubbed fingers, remittent fever, hepatosplenomegaly, and basal ganglia calcifications. Here we are reporting a case of NNS in an 11-year-old girl, who lives in eastern Algeria, born from a first-degree consanguineous marriage, she presented with erythematous patches on her face and her back, nodular erythema on her neck, swollen and painful fingers with acrocyanosis and recurrent fever that mainly occurred in cold weather. The patient received long-term treatment with low-dose glucocorticoids, along with immunomodulatory drugs (hydroxychloroquine with methotrexate), partial improvement clinically and biologically was observed. Colchicine was added to her treatment, with increased prednisone doses when she recently developed an AA amyloidosis. Our patient was diagnosed clinically with a probable NNS because she exhibited six of the eight characteristics. To the best of our knowledge, this is the first case of NNS in Africa.

## BACKGROUND

Nakajo-Nishimura syndrome (NNS) is a hereditary autoinflammatory disorder (OMIM #256040) caused by an autosomal recessive homozygous mutation of the PSMB8 gene, which encodes the immunoproteasome subunit beta 5i.^1^ NNS is classified as a proteasome-associated autoinflammatory syndrome (PAAS) with CANDLE syndrome (chronic atypical neutrophilic dermatosis with lipodystrophy and elevated temperature) and JMP syndrome (joint contractures, muscular atrophy, microcytic anemia, and panniculitis-associated lipodystrophy). The clinical manifestations of NNS are mainly pernio-like skin rashes, nodular erythema, lipodystrophy (especially on the face and upper extremities), clubbed fingers, remittent fever, hepatosplenomegaly, and basal ganglia calcifications. NNS was first described by Dr. Nakajo^2^ in 1939 in a pair of siblings (a brother and his sister) who showed secondary hypertrophic osteoperiostitis with pernio. In 1950, Dr Nishimura reported three similar cases and proposed that this was a hereditary disorder.^3^ Up to now, 30 cases have been reported, mainly from Japan and Europe. Here, we describe a case of NNS from Algeria that appears to be the first African case of NNS.

## CASE PRESENTATION

**Figure 1.**
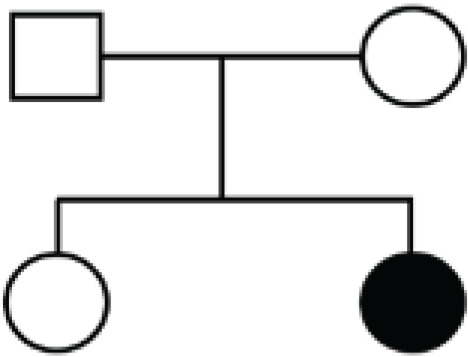
Pedigree chart of the patient.

An 11-year-old girl, who lives in eastern Algeria and is the younger of two children (her sibling being a 15-year-old healthy sister) born from a first-degree consanguineous marriage, presented from 20 days after birth with erythematous patches on her face and her back and recurrent fever that mainly occurred in cold weather. There was no family history of similar symptoms (**Figure 1**). These symptoms typically disappeared after 3 weeks and reappeared on exposure to cold weather. At the age of one year, she complained of nodular erythema on her neck, swollen and painful fingers with acrocyanosis, and multiple papules on the soles of the feet and arthralgia, which may have interfered with her ability to walk (the patient had begun to walk at the age of 3 years). Moreover, she had mental retardation that became apparent once she started to study, that is, at the age of 6 years.

**Figure 2.**
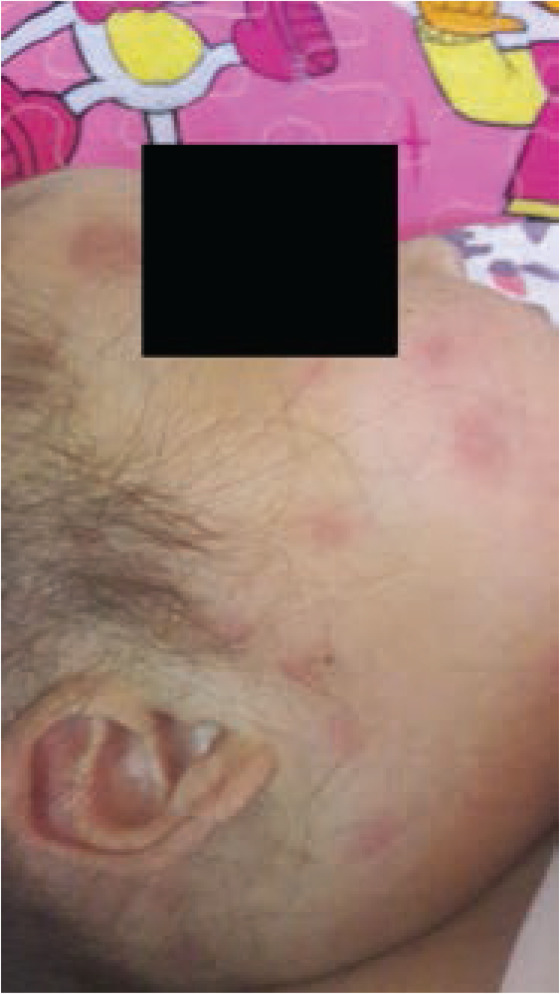
Erythematous multiple pinky patches on the face.

**Figure 3.**
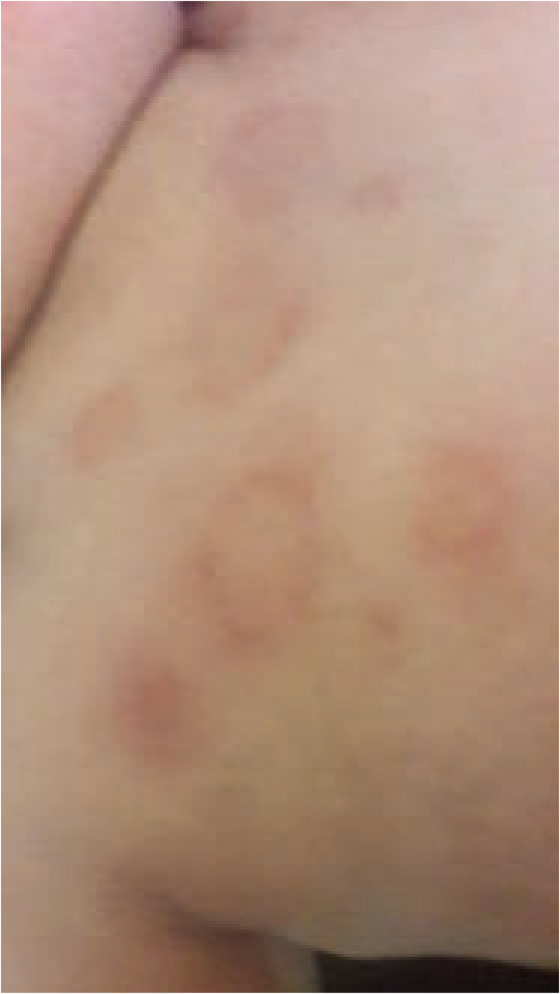
Nodular annular erythema.

**Figure 4.**
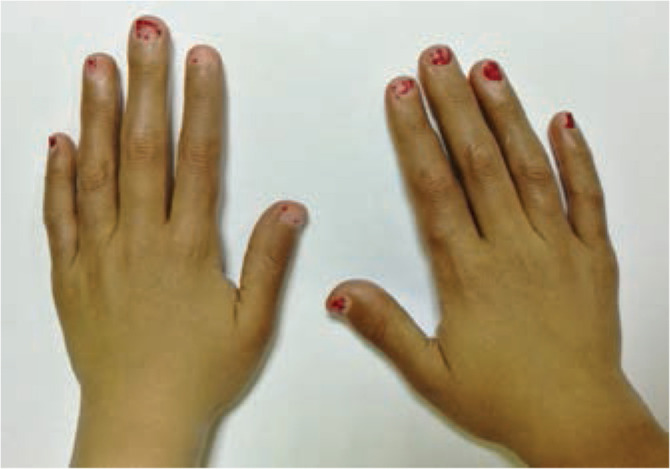
Long clubbed fingers.

Physical examination revealed that she was small in stature: she weighed 25 kg (−4.9 SD) and was 114 cm tall (−2.23 SD). Her BMI was 19.23 kg/m^2^, and she had fever (temperature, 38.7°C) at the time of admission. Multiple erythematous patches (**Figure 2**) and nodular annular erythema (**Figure 3**) were observed all over her body, especially on her extremities, and lipodystrophy was observed mainly in the upper extremities. The colour of the patches on her skin was purple to pink, and she had clubbed swollen fingers (**Figure 4**). She reported that when exposed to cold weather, she developed acrocyanosis, arthralgia, and difficulty in walking. Laboratory studies showed the following results: WBC count, 14.7 × 10^3^/mm^3^; hypochromic microcytic anaemia with a haemoglobin level of 8.1 g/dl; mean corpuscular volume, 73 fl; platelet count, 68 × 10^3^/mm^3^. Other laboratory tests revealed an ESR of 56 mm/h, C-reactive protein level of 20 mg/l, and elevated alpha- and gamma-globulin levels. The values of the autoimmunity parameters cryoglobulin, transglutaminase antibodies, rheumatoid factors, antinuclear antibodies, and anti-cyclic citrullinated peptide were within the normal range. Histopathological examination of a skin lesion under a light microscope was indicative of mastocytosis, but the immunostaining test for mastocytosis was negative. Ultrasound examination revealed homogeneous hepatomegaly and splenomegaly.

Treatment with iron and vitamin C supplements for four months did not correct her anaemia. Therefore, the patient received long-term treatment with low-dose glucocorticoids, along with immunomodulatory drugs (hydroxychloroquine along with methotrexate). Partial improvement in arthralgia and skin lesions was observed, and biologically with improvement in the level of her pro-inflammatory proteins. Unfortunately, four years after diagnosis, the patient developed AA amyloidosis, with massive proteinuria and amyloid deposits observed on renal biopsy. Colchicine was added to her treatment, and the prednisone dose was increased. Tocilizumab was not prescribed because of recurrent urinary and respiratory infections.

## DISCUSSION

The patient in the present case was clinically diagnosed with NNS. When a patient exhibits five of the eight known clinical characteristics of NNS, a definite diagnosis can be made even in the absence of genetic tests.^4^ Accordingly, our patient was diagnosed with NNS because she exhibited six of the eight characteristics: autosomal recessive heritability (as she was the child of a consanguineous marriage), recurrent remittent fever, nodular erythema with strong infiltration that intermittently appeared and disappeared, localised lipomuscular dystrophy (in the upper limbs), elongated clubbed fingers, and hepatosplenomegaly. To the best of our knowledge, this is the first case of NNS in Africa.

NNS is due to mutations in the PSMB8 gene, coding for immunoproteasomes, which are involved in the regulation of innate immune response by helping immunology cells to recognize foreign bodies carrying foreign proteins from own proteins. Mutations in NNS induce a decrease in the amount of proteins produced by the PSM8 gene, triggering a decrease in proteosomes that can damage many organs and tissues and leading to a more or less complete disease.^5^ NNS, like other autoinflammatory syndromes, can cause AA amyloidosis through chronic inflammation; this may constitute a major cause of death.^6^

There are no gold standards for the treatment of NNS. When our patient was treated with systemic corticosteroids, the fever and skin rash were alleviated. However, this effect only lasted for 3 to 4 weeks, as the lesions and fever reappeared when corticosteroid treatment was discontinued. Thus, corticosteroid treatment had no preventive effect on the occurrence of NNS symptoms. Methotrexate and calcineurin inhibitors and tocilizumab (an anti-IL-6 receptor antibody) have been found to have partial efficacy in the treatment of NNS.^7,8^ Further, baricitinib, a JAK1/2 inhibitor, was used in 10 patients with CANDLE syndrome for a mean duration of 3 years, and was found to be effective in more than 80% of the patients, with 50% achieving remission even without corticosteroid treatment.^9,10^ Additionally, treatment with ruxolib has shown certain benefits in terms of reducing symptoms as well as the IFN score in CANDLE patients,^11^ and the proteasome inhibitor bortezomib has been widely used to treat multiple myeloma and even some cases of NNS. However, some results indicate that inhibiting immunoproteasome can induce inflammatory reactions under some circumstances.^12^ Thus, based on the findings in the present case and previous reports, it might seem that our case is the first reported African case of NNS.

## References

[1] Kanazawa N. Nakajo-Nishimura syndrome: an autoinflammatory disorder showing pernio-like rashes and progressive partial lipodystrophy. Allergol Int 2012;61(2):197–206. doi: 10.2332/allergolint.11-RAI-0416. 22441638

[2] Nakajo A. Secondary hypertrophic osteoperiostosis with pernio. J Dermatol Urol 1939;45:77–86

[3] Nishimura N Deki T Kato S. Hypertrophic pulmonary osteoarthropathy with pernio-like eruption in the two families. J Dermatol Venereol 1950;60:136–41.

[4] Ohmura K. Nakajo-Nishimura syndrome and related proteasome-associated autoinflammatory syndromes. J Inflamm Res 2019;12:259–65. Published 2019 Sep 17. doi:10.2147/JIR.S194098 31576159 PMC6765212

[5] Kitamura A Maekawa Y Uehara H Izumi K Kawachi I Nishizawa M A mutation in the immunoproteasome subunit PSMB8 causes autoinflammation and lipodystrophy in humans. J Clin Invest 2011 Oct;121(10):4150–60. doi: 10.1172/JCI58414. 21881205 PMC3195477

[6] Sangiorgi E Rigante D. The Clinical Chameleon of Autoinflammatory Diseases in Children. Cells 2022 Jul 18;11(14):2231. doi: 10.3390/cells11142231. 35883675 PMC9318468

[7] Kunimoto K Ozaki F Furukawa F Kanazawa N. Beneficial effect of methotrexate on a child case of Nakajo-Nishimura syndrome. J Dermatol 2019;46(10):e365–7. doi: 10.1186/s12881-020-01060-8 31058345

[8] Cavalcante MP Brunelli JB Miranda CC Tavares MIB. CANDLE syndrome: chronic atypical neutrophilic dermatosis with lipodystrophy and elevated temperature-a rare case with a novel mutation. Eur J Pediatr 2016;175:735–740. doi:10.1007/s00431-015-2668-4 26567544

[9] Sanchez GAM Reinhardt A Ramsey S Wittkowski H Hashkes PJ Berkun Y JAK1/2 inhibition with baricitinib in the treatment of autoinflammatory interferonopathies. J Clin Invest 2018;128:3041–52. doi:10.1172/JCI98814 29649002 PMC6026004

[10] Kim H Brooks KM Tang CC Wakim P Blake M Brooks SR Pharmacokinetics, pharmacodynamics, and proposed dosing of the oral JAK1 and JAK2 inhibitor baricitinib in pediatric and young adult CANDLE and SAVI patients. Clin Pharmacol Ther 2018;104:364–73. doi:10.1002/cpt.936 29134648 PMC6089664

[11] de Jesus AA Brehm A VanTries R Pillet P Parentelli AS Montealegre Sanchez GA Novel proteasome assembly chaperone mutations in PSMG2/PAC2 cause the autoinflammatory interferonopathy CANDLE/PRAAS4. J Allergy Clin Immunol 2019;143(5):1939–43.e8. doi:10.1016/j.jaci.2018.12.1012 30664889 PMC6565382

[12] Arima K Kinoshita A Mishima H Kanazawa N Kaneko T Mizushima T Proteasome assembly defect due to a proteasome subunit b type 8 (PSMB8) mutation causes the autoinflammatory disorder, Nakajo-Nishimura syndrome. Proc Natl Acad Sci U S A 2011;108:14914–9. doi:10.1073/pnas.1106015108 21852578 PMC3169106

